# Rational Engineering of a Sub-Picomolar HIV-1 Blocker

**DOI:** 10.3390/v14112415

**Published:** 2022-10-31

**Authors:** Massimiliano Secchi, Luca Vangelista

**Affiliations:** 1 Protein Engineering and Therapeutics Group, Department of Immunology, Transplantation and Infectious Diseases, IRCCS Ospedale San Raffaele, 20132 Milan, Italy; 2 DNA Enzymology and Molecular Virology Unit, Institute of Molecular Genetics, National Research Council, 27100 Pavia, Italy; 3 Department of Biomedical Sciences, School of Medicine, Nazarbayev University, Nur-Sultan 010000, Kazakhstan

**Keywords:** HIV-1, CCR5, CCL5, protein engineering, entry inhibitor, drug combination

## Abstract

With the aim of rationally devising a refined and potent HIV-1 blocker, the cDNA of CCL5 5p12 5m, an extremely potent CCR5 antagonist, was fused to that of C37, a gp41-targeted fusion inhibitor. The resulting CCL5 5p12 5m-C37 fusion protein was expressed in *E. coli* and proved to be capable of inhibiting R5 HIV-1 strains with low to sub-picomolar IC_50_, maintaining its antagonism toward CCR5. In addition, CCL5 5p12 5m-C37 inhibits R5/X4 and X4 HIV-1 strains in the picomolar concentration range. The combination of CCL5 5p12 5m-C37 with tenofovir (TDF) exhibited a synergic effect, promoting this antiviral cocktail. Interestingly, a CCR5-targeted combination of maraviroc (MVC) with CCL5 5p12 5m-C37 led to a synergic effect that could be explained by an extensive engagement of different CCR5 conformational populations. Within the mechanism of HIV-1 entry, the CCL5 5p12 5m-C37 chimera may fit as a powerful blocker in several instances. In its possible consideration for systemic therapy or pre-exposure prophylaxis, this protein design represents an interesting lead in the combat of HIV-1 infection.

## 1. Introduction

In the fight against HIV-1 infection, the search for new compounds aimed at improving existing therapies or developing preventive approaches has identified CCR5 (the major HIV-1 co-receptor) as a relevant target [1]. CCR5 is a chemokine receptor belonging to the G protein-coupled receptor (GPCR) superfamily. When infecting target cells, HIV-1 exerts a complex entry involving several protein partners on both membranes (virus and host), ultimately leading to membrane fusion. HIV-1 entry complex dynamics, coupled to the many different molecular strategies put in place by the virus to counteract the host immune response, reflect the huge difficulties encountered in vaccine development. In spite of this, promising trials that attempt to elicit broadly neutralizing antibodies (bnAbs) are under way [2], including mRNA-based vaccines [3].

During virus entry, CCR5 engagement by HIV-1 facilitates the dislocation of CD4-bound gp120, making space for gp41 to unleash the fusion machinery [4]. HIV-1 entry is the obvious focus for the development of numerous blockers, including bnAbs and their engineered derivatives [5]. Among the CCR5 antagonists, maraviroc (MVC), a small chemical compound, and leronlimab, a monoclonal antibody, represent the most advanced therapeutical options [6,7]. CCL5, a chemokine ligand of CCR5, has been extensively engineered to create potent HIV-1 blockers acting as CCR5 antagonists [8,9,10,11]. CCL5 5p12 5m is so far the most potent CCL5 mutant reported in vitro [10] and further attempts to increase potency are under way [11]. Interestingly, CCR5 antagonists, and thus potent CCL5 derivatives, could be employed in the fight against several pathologies beyond HIV-1 infection, in which CCR5 plays a central role [12,13,14].

In the absence of a vaccine and a cure for HIV-1, pharmacological advances with new, more potent, and innovative leads are always needed. Inspired by a previous report [15], we decided to fuse CCL5 5p12 5m to the fusion inhibitor C37 [16] in order to obtain a cocktail of HIV-1 blockers embedded in the same molecule, possibly achieving a synergic effect. Other strategies were devised to achieve similar aims, such as the fusion of a small chemical compound directed against CCR5 with a gp41-targeting peptide [17], the fusion of an anti-CCR5 monoclonal antibody with two identical fusion inhibitor peptides [18], and the simultaneous targeting of gp120 and CCR5 by a bifunctional chemical compound [19], in a general view of dual targeting as an alternative to combination pharmacology [20].

We report here a CCL5 5p12 5m-C37 chimera that presents a synergic anti-HIV-1 effect over the individual components (CCL5 5p12 5m and C37) and entered the IC_50_ femtomolar scape, thus reaching an unprecedented level in anti-HIV-1 activity in vitro. CCL5 5p12 5m-C37 inhibits R5 (clade B and C), X4 and dual tropic R5/X4 HIV-1 strains. Combinations of CCL5 5p12 5m-C37 with tenofovir (TDF) and, separately, maraviroc (MVC), led to a several-fold synergic increase of antiviral activity. Analyzing the dynamics of HIV-1 entry [21], a variety of different molecular scenarios are conceivable that would allow the CCL5 5p12 5m-C37 chimera to fully exert its blockade of the virus protein machinery.

## 2. Materials and Methods

### 2.1. Drugs, Bacterial and Viral Strains

MVC (Pfizer) and TDF (GILEAD) tablets (150 mg and 300 mg, respectively) were dissolved in sterile water and used for in vitro assays as described [10,22]. The pET SUMO-CCL5 5p12 5m-linker-C37 plasmid was constructed and expanded in *E. coli* Mach1-T1 and transformed in *E. coli* BL21 (DE3) for protein expression and purification (Champion pET SUMO protein expression system, Invitrogen, Carlsbad, CA, USA) [10]. R5 HIV-1_BaL_ was obtained from the NIH Reagent Program. R5 HIV-1_5513_ and HIV-1_98IN007_ primary isolates, R5/X4 HIV-1_92US007_ and X4 HIV-1_IIIB_ viruses were kindly provided by Gabriella Scarlatti. HIV-1 strains were propagated in phytohemagglutinin-stimulated PBMCs, and the culture supernatants were harvested and stored at −80 °C until use.

### 2.2. Plasmid Construction and Protein Preparation

The plasmid necessary for the expression of the fusion protein CCL5 5p12 5m-C37 was obtained from plasmids pET28 SUMO-5p12-RANTES-linker-C37 [15] and pET SUMO-CCL5 5p12 5m [10]. BamHI restriction sites were introduced at the 3′ terminus of the CCL5 5p12 5m sequence in pET SUMO-CCL5 5p12 5m and inside the (GGGGS)_2_ linker sequence of pET28 SUMO-5p12-RANTES-linker-C37 by using QuikChange site-directed mutagenesis (Stratagene, San Diego, CA, USA) and specific primers CCL5 5p12 5m forward (5′-AATAGTCTTAGCATGAGCGGTGGTGGTGGATCCTAGAGACAAGCTTGCGG-3′), CCL5 5p12 5m reverse (5′-CCGCAAGCTTGTCTCTAGGATCCACCACCACCGCTCATGCTAAGACTATT-3′), linker-C37 forward (5′-ATGAGCGGTGGTGGTGGATCCGGTGGTGGTGGT-3′), and linker-C37 reverse (5′-ACCACCACCACCGGATCCACCACCACCGCTCAT-3′), respectively. Both plasmids were then BamHI/PvuI digested and the linker-C37 fragment excised from pET28 SUMO-5p12-RANTES-linker-C37 was ligated to the linearized pET SUMO-CCL5 5p12 5m to obtain the final expression vector pET SUMO-CCL5 5p12 5m-linker-C37. The final construct was verified by DNA sequencing.

The N-terminally SUMO tagged CCL5 5p12 5m-C37 was purified following the previously reported procedure [10,15]. Briefly, 1 liter of transformed *E. coli* BL21 (DE3) culture was induced with 1 mM IPTG at 37 °C overnight and, after inclusion bodies solubilization, the protein was refolded overnight with 90 mL of folding buffer (50 mM NaCl, 20 mM Tris-HCl, pH 8.0) supplemented with 10 mM β-mercaptoethanol at 4 °C. Then, the centrifuged supernatant was dialyzed against 4 liters of folding buffer and passed through 2 mL Ni-NTA affinity resin (Qiagen, Valencia, CA, USA). The protein was eluted with imidazole gradient (from 20 to 200 mM) in folding buffer and protein concentration evaluated by 13% SDS-PAGE stained with Coomassie brilliant blue R-250 (Applichem, Darmstadt, Germany). SUMO-tagged purified CCL5 5p12 5m-C37 was dialyzed in 4 liters of 50 mM NaCl, 20 mM Tris-HCl, pH 7.2, to remove imidazole and the SUMO tag was removed by 2 h incubation at 37 °C with recombinant yeast ULP1 protease. ULP1 protease was produced and purified as described [10,15]. Purified CCL5 5p12 5m-C37 was separated from the SUMO tag by using 0.5 mL Ni-NTA resin and collected in the flow through for protein quantification. CCL5, CCL5 5m, CCL5 5p12 5m, and CCL5 6p4 5m were obtained by using the same protocol, as previously reported [10].

### 2.3. CCL5 Derivatives Quantification

CCL5 variants concentration was evaluated by human CCL5/RANTES ELISA (DuoSet R&D Systems, Minneapolis, MN, USA), by 13% SDS-PAGE stained with Coomassie brilliant blue R-250 and by Western blot analysis by using ImageJ software. Western blot was performed by using Protran 0.2 μm BA-83 nitrocellulose membrane (Schleicher & Schuell, Keene, NH, USA) incubated overnight with polyclonal rabbit anti-human CCL5 antibody (1:1000) (PeproTech, Rocky Hill, NJ, USA), followed by 1 h incubation with horseradish peroxidase-conjugated polyclonal goat anti-rabbit antibody (1:5000) (Sigma, St. Louis, MO, USA). Chemiluminescent signals were developed by using the ECL reagent (GE Healthcare Amersham, Little Chalfont, UK).

### 2.4. Cytofluorimetry Analysis

Cytofluorimetry analysis of surface CCR5 was conducted on 1 x 10^5^ CHO-CD4-CCR5 cells incubated 4 h at 37 °C in DMEM without FBS in the presence of CCL5, MVC or the CCL5 variants (100 nM each) as previously reported [10,22]. After treatment, cells were washed twice with cold PBS containing 2% FBS and fixed with 2% fresh formaldehyde (Sigma, St. Louis, MO, USA) for 15 min. Then, washed cells were incubated with the 3A9 anti-CCR5 monoclonal antibody (BD Bioscience, San Jose, CA, USA) and donkey anti-mouse IgG (H+L) Alexa Fluor 488 (Invitrogen, Carlsbad, CA, USA) as described [10,20] for 15 min at 4 °C. After washing, cells were analyzed by using the Gallios Flow Cytometer (Beckman Coulter Inc., Brea, CA, USA) and data was analyzed by using the FlowJo software (Tree Star Inc., Ashland, OR, USA).

### 2.5. HIV-1 Infection Assay

Acute HIV-1 infection was obtained by adding the different HIV-1 strains (50 median tissue culture infectious doses, TCID_50_/well) to PM1 cells [23] (2 × 10^4^/well) in complete RPMI medium. Experiments were performed in triplicate by using 96-well round-bottom microtiter plates in the presence or absence of inhibitors and virus replication was assayed at day 4 post-infection by the p24 antigen ELISA as previously described [22]. Human monocyte cultures were established from peripheral blood mononuclear cells (PBMC) isolated from Ficoll–Hypaque (Pharmacia, Uppsala, Sweden) density gradient centrifugation of buffy coat preparations obtained from healthy HIV-1-seronegative blood donors. The sampling was performed according to the Declaration of Helsinki, and approval was given by the Institutional Review Board named “Comitato Etico della Fondazione San Raffaele del Monte Tabor, Milan, Italy” (protocol no 95/DG). All anonymized subject provided written informed consent, and all methods were performed in accordance with the relevant Italian guidelines and regulations. Human monocyte-derived macrophages (MDM) were obtained after 7–10 days of monocyte differentiation and infected in quadruplicate with HIV-1_BaL_ (50 TCID_50_/well) in the presence or absence of inhibitors as described [22]. After overnight incubation, the unbound virus was removed by extensive washing, fresh medium was added, and cultures were further incubated at 37 °C. Supernatants were harvested at day 4 for p24 antigen determination.

### 2.6. Combinations and Statistical Analysis

Experimental design and analysis of synergy, additivity, or antagonism between different compounds were based on the combination index (CI) method of Chou and Talalay [24,25]. In HIV-1 infection assays, each compound was tested individually and in a fixed molar ratio (IC_50_:IC_50_) combination over a range of two-fold serial dilutions. The 50%, 75%, and 90% combination indices (CI_50_, CI_75_, and CI_90_) to determine the effect of the interactions between the compounds were calculated by using the Calcusyn software 2.0 (Biosoft, Cambridge, UK) [22]. A CI of <0.9 indicates synergy, a CI from 0.9 to 1.1 indicates additivity, and a CI of >1.1 indicates antagonism. Dose-response curves were fitted by using GraphPad Prism version 5.04 (GraphPad Software, La Jolla, CA, USA) in order to calculate IC_50_ concentrations through nonlinear regression analysis. All data are expressed as the means ± SD for two independent experiments performed in triplicate. All p-values were combined according to the Fisher’s method.

## 3. Results

### 3.1. Design and Production of the CCL5 5p12 5m-C37 Chimera

**Figure 1:**
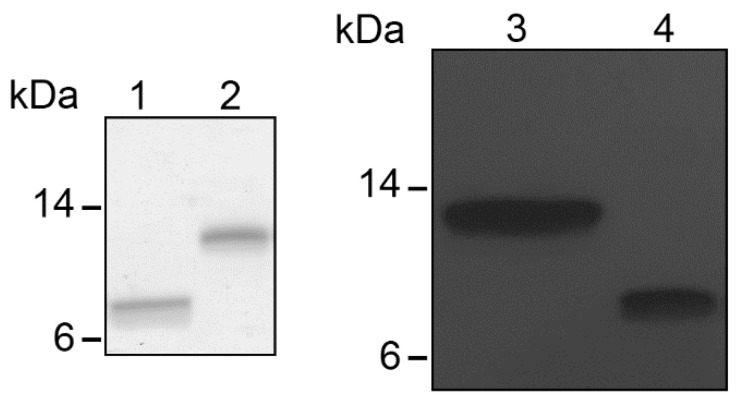
Purified CCL5 5p12 5m-C37. Left panel: Coomassie blue-stained SDS-PAGE of CCL5 5p12 5m (lane 1) and CCL5 5p12 5m-C37 (lane 2) purified from recombinant *E. coli* expression. CCL5 5p12 5m and CCL5 5p12 5m-C37 have an expected molecular mass of 7.8 and 13 kDa, respectively. Right panel: Western blot of purified CCL5 5p12 5m-C37 (lane 3) and CCL5 5p12 5m (lane 4).

We previously described the high anti-HIV-1 activity of CCL5 5p12 5m [10]. Acting as a CCR5 antagonist thorough its N-terminus, this molecule potently inhibited different HIV-1 R5 strains with low picomolar IC_50_. Despite this high antiviral activity, the entry of X4 viruses could obviously not be blocked. The peptide C37 is a well-characterized fusion inhibitor active at nanomolar concentration against different HIV-1 strains, including X4 viruses [26]. C37 is a peptide deriving from the C-terminal moiety of gp41 ectodomain and acts by binding to the transient pre-hairpin intermediate on the N-terminal portion of gp41 ectodomain, thus preventing the assembly of gp41 trimer of hairpins and blocking membrane fusion [16]. Inspired by the successful production of N-terminally modified CCL5-C37 chimeric inhibitors capable to inhibit both R5 and X4 tropic HIV-1 strains [15], the C-terminus of CCL5 5p12 5m was fused to C37, interspaced by the selected linker (GGGGS)_2_ [15] and the protein was denoted CCL5 5p12 5m-C37. The resulting chimera of about 27 kDa was expressed in *E. coli* as N-terminally 6His SUMO tagged, it formed inclusion bodies and was refolded and easily purified to homogeneity after removal of the SUMO tag by using the ULP1 protease, according to previous reports [10,15]. Purified CCL5 5p12 5m-C37 was analyzed by SDS-PAGE and quantified by Western blot (Figure 1).

In contrast to N-terminally modified CCL5 variants, CCL5 5p12 5m adds to the 5p12 N-terminal modification (switching CCL5 to a CCR5 antagonist) [8] five extra mutations (5m) that encompass CCL5 core stabilization, monomerization and an increased CCR5 active site occupancy [10,11], thus accounting for its superior anti-HIV-1 activity in vitro [10]. CCL5 5p12 5m-C37 was designed with the rationale to achieve HIV-1 blocking activity at sub-picomolar concentration.

### 3.2. Verification of CCR5 Antagonism by CCL5 5p12 5m-C37

CCL5 C-terminus does not contribute to the interaction with CCR5, regardless of agonist or antagonist activity exerted by CCL5 variants [9,27,28]; therefore, and as already proven [15], the C-terminus was the ideal site for the fusion with C37. The CCR5 antagonist property of CCL5 5p12 5m was thus verified in the CCL5 5p12 5m-C37 chimera. Cytofluorimetric analysis of CCR5 internalization upon engagement by several CCR5 ligands demonstrated that CCL5 5p12 5m-C37 preserved its crucial function as CCR5 antagonist.

**Figure 2:**
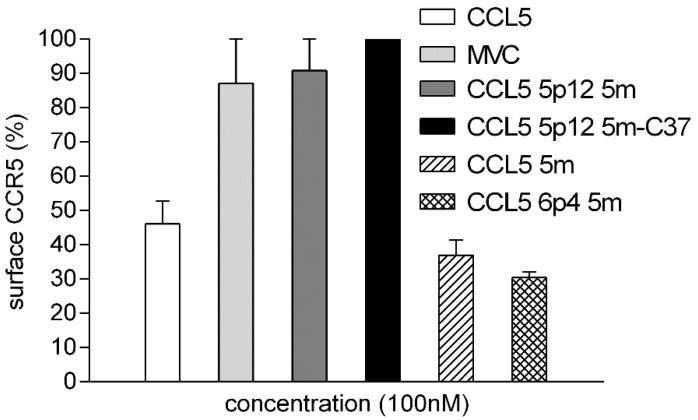
CCR5 internalization by CCL5 variants and MVC. Cytofluorimetry of CHO-CD4-CCR5 cells labeled with the anti-CCR5 mAb 3A9. Cell surface CCR5 was set at 100% for cells treated with 3A9. Surface CCR5 of cells pre-incubated with 100 nM of CCL5, CCL5 5m, CCL5 6p4 5m, CCL5 5p12 5m, MVC, and CCL5 5p12 5m-C37 is shown as the mean ± SD of cell surface CCR5 expressed as percentage of control and is representative of two independent experiments.

As indicated in Figure 2, the antagonism of CCL5 5p12 5m was maintained after fusion to the flexible linker and the C37 peptide. CCL5 5p12 5m and MVC were used as control antagonists, whereas CCL5, CCL5 5m, and CCL5 6p4 5m were used as agonist controls and led to different extent of receptor internalization (Figure 2). CCL5 5m and CCL5 6p4 5m are potent CCR5 agonists that incorporate the same five mutations inserted in CCL5 5p12 5m and induce higher receptor internalization as compared to CCL5 [10]. Indeed, the 5m mutations lead to a potent agonist when the CCL5 N-terminus is not modified and to a very potent agonist when the 6p4 N-terminus is inserted [10].

### 3.3. Anti-HIV-1 Activity by CCL5 5p12 5m-C37

The antiviral potency of CCL5 5p12 5m-C37 was tested on different HIV-1 strains, clades, and cell types, in order to cover for its activity spectrum. CCL5 5p12 5m was used as potency control counterpart. The anti-HIV-1 activity of C37 is in the low nanomolar IC_50_ range, and C37 equimolar combination with potent CCL5 derivatives (acting at low picomolar IC_50_) as separate molecular entities led to a negligible addition effect by C37, as expected [15].

**Figure 3:**
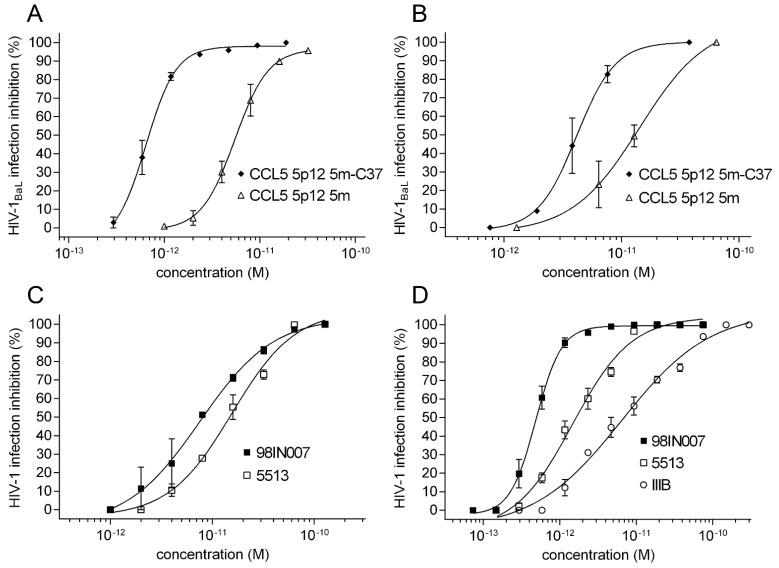
Anti-HIV-1 activity of CCL5 5p12 5m-C37. (**A**) Inhibition of R5 tropic laboratory-adapted clade B HIV-1_BaL_ by CCL5 5p12 5m-C37 tested by acute infection in PM1 cells and measured by a p24-based assay after 4 days of infection. (**B**) HIV-1_BaL_ inhibition of CCL5 5p12 5m-C37 tested by acute infection assay as in (**A**) in MDM. CCL5 5p12 5m was used as control. (**C**) CCL5 5p12 5m inhibition of R5 tropic primary HIV-1 strains 5513 (clade B) and 98IN007 (clade C) as in (**A**). (**D**) CCL5 5p12 5m-C37 inhibition of R5 tropic primary HIV-1 strains 5513 (clade B) and 98IN007 (clade C) and X4 tropic laboratory adapted strain IIIB as in (**A**). Values indicate the means ± SD of two independent experiments performed in triplicate.

Acute HIV-1_BaL_ (R5 tropic) infection inhibition was tested by using a p24-based assay in PM1 cells and MDM (Figure 3A,B), similarly to previous reports [10,29]. Although the low picomolar IC_50_ of CCL5 5p12 5m was confirmed, the IC_50_ of CCL5 5p12 5m-C37 improved 8.5- and 3.5-fold in PM1 cells and MDM, respectively, reaching 0.64 pM for PM1 cells (Table 1). In a p24-based assay on PM1 cells, C37 inhibited HIV-1_BaL_ with a 15 nM IC_50_, similarly to previously reported data [15]. CCL5 5p12 5m-C37 also showed higher IC_50_ against primary R5 tropic HIV-1 strains of clade B (5513) and C (98IN007) in PM1 cells. Of note, the potency of the chimera against 98IN007 reached a 480 fM IC_50_. Unlike CCL5 5p12 5m, its C37 chimeric form blocked PM1 cells infection by dual tropic R5/X4 (92US077) and X4 tropic (IIIB) HIV-1 strains (Figure 3C,D and Table 1).

Zhao et al. [15] found that in CXCR4^+^ cells, where CCR5 is absent, the residual HIV-1 inhibitory activity of their CCL5 derivative-C37 chimeras is identical to that of C37 alone, as expected. Similar results were obtained when CCR5^+^ CXCR4^+^ cells were preincubated with the CCL5 variant corresponding to that used in the chimera [15].

### 3.4. CCL5 5p12 5m-C37 Drug Combinations

**Figure 4:**
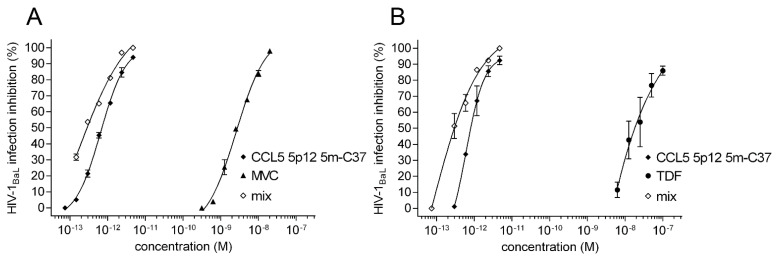
CCL5 5p12 5m-C37 drug combinations. HIV-1_BaL_ inhibition was evaluated by acute infection assays as described in Figure 3A. CCL5 5p12 5m-C37 was tested in combination with MVC (**A**) and TDF (**B**). The dose-response curves of mixed inhibitors (mix) are referred to CCL5 5p12 5m-C37 (*p*-values < 0.0001). Values indicate the means ± SD of two independent experiments performed in triplicate.

The effect of the combination of CCL5 5p12 5m-C37 with the CCR5 antagonist MVC and, separately, with TDF (a nucleotide analogue reverse transcriptase inhibitor) was investigated in acute infection inhibition experiments (Figure 4A,B). Both combinations potently inhibited HIV-1_BaL_ infection in PM1 cells giving synergic effects of CI_50_ 0.78 ± 0.05 and 0.85 ± 0.02 with MVC and TDF, respectively (Table 2). The IC_50_ of CCL5 5p12 5m-C37 improved 4-fold with MVC and 9.6-fold with TDF, fully entering the femtomolar scape (Table 3). The IC_50_ mix values for TDF and MVC are 1.44 nM and 1.07 nM, respectively. These combinations prove that CCL5 5p12 5m-C37 potency is enhanced in a synergic manner, further attesting its potential usage in anti-HIV-1 treatments.

## 4. Discussion

The HIV-1 pandemic has afflicted humanity for more than 30 years, and it is far from being resolved. Given the fact that a vaccine and a cure for HIV-1 infection are still a remote hope, new therapeutic and preventative measures may improve the life of infected individuals. CCR5 is an important target for HIV-1 infection therapy and prevention. We recently developed CCL5 5p12 5m, a potent CCR5 antagonist based on novel amino acid substitutions within the core of CCL5 and the adoption of a known N-terminal modification [10]. In this work, the fusion of CCL5 5p12 5m with C37 led to a synergic increase in the overall anti-HIV-1 activity of the chimeric protein.

**Figure 5:**
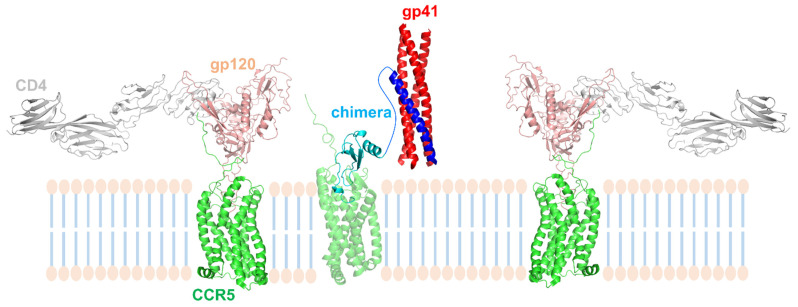
Model of the synergic inhibitory effect exerted by CCL5 5p12 5m-C37 on R5 tropic HIV-1 strains. Spare molecules of CCR5 escape inhibition by the CCL5 5p12 5m moiety of the chimera and allow CD4-bound gp120 to engage them, making space for gp41 to undergo the conformational changes leading to membrane fusion. The membrane fusion machinery of gp41 is readily blocked by the membrane proximity (provided by chimera-bound CCR5 molecules) of the C37 moiety of the chimera. Three-dimensional ribbon representation were generated by using PyMOL: CD4 (grey):gp120 (salmon):CCR5 (green) complexes (left and right) from PDB entry 6MET [4]; gp41 N-heptad repeat (red):C34 (blue) (arbitrarily represented here as C37) complex from PDB entry 6R2G [42]; CCR5 (transparent green):CCL5 5p12 5m (light blue) complex was modeled [11] upon PDB entry 5UIW [9]. The linker in the CCL5 5p12 5m-C37 chimera is arbitrarily shaped in blue. The cell membrane bilayer is schematized.

The union of a CCR5 antagonist with a fusion inhibitor covers two major steps of HIV-1 entry blockade. The kinetics of coreceptor engagement and gp41-mediated membrane fusion is possibly allowing the dual targeting reported here, justifying this approach, as discussed in a previous report [15]. There are a number of instances in which the molecular proximity of CCL5 5p12 5m and C37 would favor either a broader co-receptor inhibition or a more efficient R5-tropic virus inhibition. GPCRs also exist within cell membranes as homo- or heterodimers, as well as oligomers [30], and CCR5 is involved in higher molecular structures [31,32]. CCL5 5p12 5m is a highly potent inhibitor of HIV-1 entry; however, it is conceivable that, due to conformational plasticity of CCR5, spare molecules of CCR5 on the cell surface escape blockade and may be actively engaged by CD4-bound gp120, eventually leading to activation of gp41-mediated membrane fusion [33]. In such molecular instance, a CCL5 5p12 5m-C37-engaged CCR5 molecule, either in proximity or as a CCR5 dimeric partner to the gp120-bound CCR5, could provide the C37 inhibitor, readily preventing infection. Therefore, the CCL5 5p12 5m portion of the chimera may serve a dual function: act as potent HIV-1 inhibitor and refine the blockade by quenching residual virus activation of the gp41 fusion machinery. The provision of C37 in a stable close proximity to the target cell membrane, via CCL5 5p12 5m docking into CCR5 and absence of receptor internalization, most likely accounts for the observed synergic antiviral effect. We modeled the synergic inhibitory effect of CCL5 5p12 5m-C37 on R5 tropic HIV-1 strains in Figure 5. In addition, a similar molecular scenario is conceivable for X4 tropic viruses with the close membrane proximity of CCR5-bound CCL5 5p12 5m-C37 providing the fusion blockade (through readily available C37 moieties) to CXCR4 engaged HIV-1 strains.

Current pharmacological treatment of HIV-1 infection is very effective; however, a number of concerns remain. The complete elimination of HIV-1 from infected individuals is a long-sought, yet, to date, unreachable accomplishment [34]. Drug resistance and chronic pathologies associated to HIV-1 infection under lifelong drug regimen remain a burden to patients. In this scenario, new pharmacological concepts are emerging that are aimed at achieving a cure for HIV-1 infection, or to further reduce the risks associated with drug resistance. In this direction, exceedingly potent lead compounds could provide the perspective for a safer therapy, significantly reducing the risk of drug resistance. The possibility of employing CCL5 derivatives as potent anti-HIV-1 CCR5 antagonists presents a major advantage over small chemical compounds such as MVC, i.e., the virtual absence of HIV-1 resistance [35,36]. This has been illustrated by the capacity of CCL5 and its derivatives to fully engage the CCR5 active cleft, leaving no chance for HIV-1 gp120 mutational adaptation, and thus for the insurgence of resistant strains of the virus [9,27,28]. Importantly, a compound such as CCL5 5p12 5m-C37 could also be considered for topical administration as anti-HIV-1 microbicide. Indeed, CCL5 derivatives are being studied and developed as microbicides in drug formulations [37] and live microbicides from recombinant commensal bacteria delivery [10,29,38], and combinations of these with other drugs [10,39]. Distinct combinations of CCL5 5p12 5m-C37 reinforce the concept of possible new drug regimens. The combination with MVC could lead to a nearly complete biochemical blockade of CCR5, likely resembling a pharmacological knockout of the receptor with the advantage, over gene-editing knockout, to preserve CCR5 for the immune homeostasis once the treatment is ended. The combination with TDF could endorse a two-drug regimen with the advantage of reducing the use of anti-HIV-1 drugs [40], allowing for other options in the case of insurgence of resistance. High potency, efficient blockade of resistant strains, and reduction of the number of drugs are some of the features to look for in the development of new drugs and regimens for HIV-1 infection and prevention [41].

## Figures and Tables

**Table 1:** Anti-HIV-1 activity.

HIV-1 Strain	Subtype	IC_50_ CCL5 5p12 5m	IC_50_ CCL5 5p12 5m-C37
BaL (PM1)	R5, clade B	5.47 pM	0.64 pM
BaL (MDM)	R5, clade B	14.18 pM	4.13 pM
5513	R5, clade B	15.54 pM	1.47 pM
98IN007	R5, clade C	7.55 pM	0.48 pM
92US077	R5/X4	>600 pM	>80 pM
IIIB	X4	>10,000 pM	6.45 pM

**Table 2:** Combination indexes.

CCL5 5p12 5m-C37	CI_50_	CI_75_	CI_90_	CI_50_ Effect
MVC	0.78 ± 0.05	0.52 ± 0.01	0.39 ± 0.01	Synergy
TDF	0.85 ± 0.02	0.66 ± 0.02	0.57 ± 0.01	Synergy

**Table 3:** IC_50_ in combinations.

Combination	IC_50_ CCL5 5p12 5m-C37	IC_50_ MVC	IC_50_ TDF	IC_50_ Mix CCL5 5p12 5m-C37
CCL5 5p12 5m-C37 MVC	0.64 pM	2.55 nM		0.16 pM
CCL5 5p12 5m-C37 TDF	0.67 pM		12.4 nM	0.07 pM

## Data Availability

Not Applicable.
